# Relationship between Postmenopausal Estrogen Deficiency and Aneurysmal Subarachnoid Hemorrhage

**DOI:** 10.1155/2015/720141

**Published:** 2015-10-11

**Authors:** Sadaharu Tabuchi

**Affiliations:** Department of Neurosurgery, Tottori Prefectural Central Hospital, Tottori 680-0901, Japan

## Abstract

Aneurysmal subarachnoid hemorrhage (SAH) is one of the most severe forms of stroke, which results from the rupture of a cerebral aneurysm. SAH is the only type of stroke with a female predominance, suggesting that reproductive factors may play a significant role in the etiology. Estrogen has important effects on vascular physiology and pathophysiology of cerebral aneurysm and SAH and, thus, potential therapeutic implications. There have been growing bodies of epidemiological and experimental studies which support the hypothesis of a significant relationship between estrogen deficiency and cerebral aneurysm formation with subsequent SAH. This hypothesis is the focus of this review as well as possible pathology-based therapeutics with regard to aspects of molecular pathophysiology, especially related to women's health.

## 1. Introduction

Aneurysmal subarachnoid hemorrhage (SAH) is a devastating stroke subtype, which frequently occurs as the result of a ruptured cerebral aneurysm. Mortality rates for ruptured cerebral aneurysms continue to average near 50%, with 10% of patients dying before ever reaching the hospital and approximately 20% sustaining severe disability [1]. Unlike most other types of cerebrovascular disease, SAH occurs more frequently in women than in men [2]. Mortality rates of female SAH patients have been reported to be higher than those for men [3]. Women are also more likely to have multiple aneurysms than men [4]. Women had a significantly higher risk for* de novo* aneurysm formation than men in a long-term follow-up study, and being female was a significant independent risk factor for aneurysm growth [5]. Additionally, it has been shown that faster aneurysm growth increases the likelihood of rupture. Aneurysm size was also associated with a tendency to rupture. There are few reports that have specifically examined the differences in aneurysmal size between the sexes. Although Li et al. reported that the mean diameter of unruptured cerebral aneurysms (UCAs) was larger in women than in men [6], no difference was found in another report [7], and the sizes of ruptured cerebral aneurysms were not statistically significantly different between the sexes [8].

The gender-related predominance is important because many of the risk factors for cerebral aneurysm, including hypertension, cigarette smoking, and alcohol use, are generally more common in men. A systematic review suggested that the gender distribution of SAH varies with age. At younger ages, the incidence is higher in men, whereas, after the age of 55, the incidence is higher in women [9]. Furthermore, earlier age at menopause is associated with a greater risk of cerebral aneurysm [10]. The loss of estrogen earlier in a woman's life may contribute to the pathogenesis of cerebral aneurysm formation. Because the incidence of SAH is higher after menopause than before [11], it has been suggested that the sex hormones, especially estrogen, might be protective against the condition [12]. Herein is a review focused on the hypothesis of the relationship between aneurysmal SAH and estrogen deficiency along with a discussion of the therapeutic implications.

## 2. Review

### 2.1. Sex-Specific Hormonal Factors and SAH

Premenopausal women, especially those without a history of smoking or hypertension, are at a reduced risk for subarachnoid hemorrhage (SAH) compared with age-matched postmenopausal women [13]. A study of Japanese women with their first occurrence of aneurysmal SAH found that an earlier age at menarche (adjusted odds ratio, 3.24) and nulliparity (adjusted odds ratio, 4.23) were associated with an increased risk of SAH. These effects appeared to be additive, and women with both early menarche and nulliparity had a correspondingly increased risk (adjusted odds ratio, 6.37) of SAH [14]. Therefore, a sex-specific hormonal factor may play a role in the pathogenesis of aneurysm formation and rupture. The collagen wasting commonly observed in bone and skin in the postmenopausal period due to decreased estrogen levels could possibly be responsible for the formation of aneurysms in the proximal segments of the cerebral arteries, as it occurs in various connective tissue diseases [15–17]. Between the ages of 50 and 59, menopause typically occurs, and estrogen levels begin to decrease. Hemodynamics, in conjunction with progressive collagen loss in the vascular wall, appears crucial for the development of an aneurysm. In a population-based, case-control study, several hormonal factors in women were reported to alter the risk for SAH. Factors leading to a state of relative estrogen deficiency, such as menopause and the immediate perimenstrual phase of the menstrual cycle, were associated with an increased risk [18]. Conversely, hormone replacement therapy (HRT) was associated with a reduced risk for SAH [13]. As estrogen deficiency alone would not explain the higher incidence in women compared with that in men, the change in estrogen levels may be more important than the absolute levels. Men would be at less risk because they do not experience as much of a dramatic estrogen withdrawal as women. Estrogen levels markedly change during the menstrual cycle, and relative estrogen deficiency occurs in the immediate perimenstrual phase of the menstrual cycle [13]. This periodic hormonal change may promote aneurysm formation [19].

Many studies on UCAs with location-based evaluation have shown similar results suggesting that UCAs in the internal carotid artery (ICA) were most common [7, 20, 21]. Harada et al. examined the prevalence of UCAs in healthy asymptomatic Japanese adults and found that there were significant differences in the locations of UCAs by gender (*P* < 0.001). ICA involvement was more frequent in women, whereas the anterior cerebral artery (ACA) and middle cerebral artery (MCA) were more common in men [7]. Even in cases of ruptured aneurysms, the rate of occurrence in the ICA was reported to be higher in women than in men [22–25]. As the aneurysms of the ACA and MCA are more commonly found in men, it can be speculated that hemodynamic factors are more important in the formation of aneurysms in men. On the other hand, an intrinsic weakness of the vessel wall due to hormonal factors may play a major role in promoting the formation of cerebral aneurysm in female patients [24].

Chen et al. reported that exposure to exogenous estrogen agents in women is associated with a lower frequency of cerebral aneurysms [26]. The physiologic drops in estrogen and/or low endogenous levels of estrogen that occur during the menstrual cycle, and particularly at menopause, may not only play an important role in cerebral aneurysm formation but may also serve as potential therapeutic targets.

### 2.2. Cerebral Aneurysm Formation and Estrogen

Aneurysms are abnormal dilatations of vessels in the vascular system, and they exist in two major forms: saccular and fusiform. As for cerebral aneurysm, the saccular type dominates. The saccular aneurysm is a thin-walled, sphere-like expansion from the branching region of a major cerebral artery. Meanwhile, fusiform aneurysms have different etiologies (arteriosclerosis, intramural dissection, etc.) and are classified into different entities [27, 28]. Unlike saccular aneurysms, which present more often with SAH, fusiform aneurysms present more often with ischemic stroke or mass effect [29]. Thus, saccular aneurysms are the focus of this paper.

Like all arteries, cerebral arteries consist of three layers: intima (adjacent to the lumen), media, and adventitia. The internal elastic lamina separates the intima from the media and is essentially a fenestrated sheet of elastin. The media is composed of smooth muscle cells and collagen fibers. The adventitia consists of collagen fibers, elastin, fibroblasts, and the vasa vasorum. The adventitia is thought to limit acute overdistension in all vascular vessels at higher levels of pressure [30]. Collagen fibers are mainly produced by fibroblasts. Fibroblasts also produce, organize, and remove the extracellular matrix. The repair and maintenance of connective tissue are performed predominantly by fibroblasts. The loss of media in the location of aneurysms is taken to be responsible for the initiation of aneurysm growth. The aneurysm is regarded as a development of the adventitia, which is composed of a layer of collagen fibers. The collagen fibers are the only load-bearing constituent in the aneurysm wall; their production and degradation depend on the stretch of the wall and are responsible for aneurysm growth [31].

Hemodynamics also play an important role in the development of aneurysms. There are three hemodynamic forces to consider. They include shear stress, dynamic blood pressure, and static blood pressure. The shear stresses are considerably higher at the apex of arterial bifurcations. This explains why cerebral aneurysm is commonly found at arterial bifurcations where excessive hemodynamic stresses are exerted on arterial walls [32].

Handa et al. reported three important factors in the formation of cerebral aneurysms in experimental animals: altered hemodynamic stress in the circle of Willis, hypertension, and increased vessel fragility. Estrogen could potentially affect all of these factors and thus influence the risk for cerebral aneurysm formation and rupture [33]. Estrogen has been found to improve lipid profiles [34, 35] and thus may reduce the risk for arteriosclerosis, which has been considered a risk factor for aneurysm formation because of altered hemodynamics. In addition, low-dose estrogens are associated with a reduced blood pressure, as is HRT [36].

It is thought that the arteries, including the internal carotid artery (ICA), could undergo postmenopausal connective tissue changes. The media layer has the highest connective tissue component, including collagen types I and III. It has been shown that menopause has similar effects on the connective tissue changes of the carotid artery media [37]. HRT has a morphological effect on the carotid arteries, encouraging thickening of the layers with the highest connective tissue component (media and adventitia) in postmenopausal women [38].

Wall thickness of a cerebral aneurysm is important for the integrity of the aneurysm wall and aneurysmal growth and rupture, which is poorly documented. Kadasi et al. reported an interesting observation about wall thickness of UCAs using intraoperative microscopy. They reported that the proportion of superthin translucent tissue at the aneurysm dome was significantly greater (*P* = 0.038) in women compared with men, suggestive of the susceptibility for rupture [39]. Estrogen might play an important role in vascular and aneurysmal integrity through the control of collagen content and wall thickness.

Taken together, it is considered that estrogen makes a significant contribution to the etiology of cerebral aneurysm formation and growth.

### 2.3. Estrogen Signaling in Cerebral Arteries and Aneurysm

Endogenous natural estrogens include estrone (E1), estradiol (E2), and estriol (E3).

In women, E2 is the main form of circulating estrogen. The ovaries are the main source of circulating E2 in premenopausal women [40]. Most E1 and E3 are formed in the liver from E2 or in peripheral tissues from androstenedione. The sources and plasma levels of estrogens change with age. In postmenopausal women, circulating androstenedione, testosterone, and E1 are the major precursors of estrogen production in peripheral tissues. E2 metabolism varies with the phase of the menstrual cycle, menopausal status, ethnic background, and gene polymorphism [41]. Changes in E2 metabolism with aging may alter its effects on the vasculature.

Estrogens bind estrogen receptors (ERs) with high affinity and specificity. There are two established genes encoding ER: ER-alpha and ER-beta. These receptors are members of the nuclear receptor superfamily and function as ligand-activated transcription factors to produce so-called genomic effects. Endothelial and vascular smooth muscle cells are replete with both ER-alpha and ER-beta receptors [42, 43]. Sex- and age-related differences in ER expression have been shown [44]. Levels of ER-alpha and ER-beta appear to be differentially regulated by estrogen itself. However, regulation of estrogen receptors may be dependent upon the tissue and duration of estrogen treatment [45].

A novel seven-transmembrane, G protein-coupled receptor, originally identified as an orphan receptor termed GPR30, has been identified [45]. This receptor was later named the G protein-coupled estrogen receptor (GPER). GPER is structurally unrelated to ER-alpha or ER-beta, binds E2 with high affinity, and mediates some of its nongenomic effects [46]. Although GPER is widely distributed in the brain and vasculature [45], its functional role needs further investigation [40].

ER-beta is a predominant form of ER in human cerebral aneurysms and cerebral arteries [47, 48]. Tada et al. reported that estrogen prevented cerebral aneurysmal growth and rupture in ovariectomized female mice, consistent with epidemiological studies [47, 48]. The protective effect of ER-beta activation for aneurysm growth and rupture is dependent on the production of nitric oxide (NO). Cardioprotective effects of estrogen can also be mediated by ER-beta in an NO-dependent manner [49].

### 2.4. Preventive and Therapeutic Potential of Hormone Replacement Therapy (HRT) in Unruptured Cerebral Aneurysms (UCAs)

#### 2.4.1. HRT and Its Problems

Estrogen regulates a number of inflammatory cascades and contributes to vascular wall integrity. The dysregulation of these mechanisms has been linked to aneurysm formation, growth, and rupture, with the strong suggestion that administration of estrogen replacement therapy could prevent this pathology. However, the negative results for beneficial effects of E2 therapy (HRT) have been reported in several randomized controlled trials (RCTs) for menopausal symptoms. The failure of HRT in RCTs is suspected to be related to age-related changes in ER number, distribution, and downstream signaling mechanisms, as well as structural changes in vasculature [40]. If estrogen is protective from menopausal symptoms but cannot reverse preexisting vascular disease, then perhaps HRT has not been administered early enough in menopause in these studies to be effective [50]. Age may be an important factor in estrogenic effects on inflammatory responses, with a loss of estrogenic efficacy in elderly women. The vascular action of estrogenic treatments suggests that timing matters [51]. Recent animal and human studies now support the “critical period” hypothesis for E2 therapy whereby E2 therapy must begin soon after the loss of endogenous E2 production to have a significant effect [52]. When administered earlier in the disease progression, estrogen may be advantageous by decreasing the likelihood of the development of cerebral aneurysm and subsequent rupture leading to SAH.

Besides HRT's beneficial effects, there is clear evidence that oral estrogens increase the risk of venous thromboembolism (VTE) among postmenopausal women [53]. The Estrogen and Thromboembolism Risk (ESTHER) study confirmed that oral estrogens increased VTE risk, whereas transdermal estrogens had little or no impact on the development of thrombosis [54]. There is increasing evidence that the timing of HRT initiation may play a crucial role in determining chronic heart disease risk among estrogen users [55]. Coronary risk tended to be lowered compared to placebo when HRT was initiated during the first 10 years after the onset of menopause. This risk, however, tended to increase when HRT was started 10 years after menopause and was further intensified after 20 years [55]. Another significant drawback is that long-term HRT may increase the risk of breast cancer and meningioma in women [56].

At present, there are many obstacles that need to be overcome prior to the actual application of conventional HRT to the prevention/treatment of cerebral aneurysm.

#### 2.4.2. Selective Estrogen Receptor Modulators (SERMs)

Subtype-specific ER agonists coupled with specific targeting or drug-delivery techniques could be useful in modulating vascular ER activity in a specific blood vessel without affecting other vessels in the systemic circulation. A number of selective estrogen receptor modulators (SERMs) have been developed [57]. SERMs could be selective in targeting vascular ERs while having few undesirable effects, such as VTE, cardiovascular disease, and breast cancer. It is conceivable that one could design SERMs that would retain most of the desired effects of E2 to targeted tissues and organs but that would be devoid of the undesirable effects of E2. SERMs confer vasoprotective effects by reducing the release of reactive oxygen species. In prior reports, the studied SERM attenuated the development of experimental aneurysms* in vivo* [58]. As superior protective effects on the development and rupture of cerebral aneurysms of selective ER-beta agonists have been reported compared to estrogen [47, 48], production of new-generation SERMs with a favorable tissue specificity profile may be promising in preventing the growth and rupture of cerebral aneurysms in postmenopausal women.

#### 2.4.3. Phytoestrogens

Phytoestrogens are estrogenic compounds of plant origin classified into different groups with structural similarities to E2 that allow them to mimic the effects of E2. Among them, isoflavones such as genistein are the most studied and are most potent phytoestrogens and are found mainly in soy bean-derived foods [59]. The effects of phytoestrogens are partly mediated via estrogen receptors: ER-alpha, ER-beta, and possibly GPER. The interaction of phytoestrogens with ERs is thought to induce both genomic and nongenomic effects in many tissues, including the vasculature. Genistein has a high affinity for ER-beta, almost identical to that of E2, while its affinity for ER-alpha is only 6% of that of E2 [59]. As clinical trials have shown negative results for vascular benefit and an increased risk of cardiovascular disease with conventional HRT [60], phytoestrogens are being considered as an alternative to conventional HRT [59].

Extracellular matrix (ECM) is a major component of the blood vessel architecture and plays an important role in the control of vascular wall integrity and vascular remodeling. Phytoestrogens affect various components of the ECM, including collagen and elastin [59]. One experimental study showed that phytoestrogens inhibit aneurysm formation in mice [61]. Further studies are needed to investigate the potential beneficial effects of phytoestrogens in preventing cerebral aneurysm formation and growth.

### 2.5. Future Perspectives for Prevention and Treatment of Unruptured Cerebral Aneurysm (UCA) in Women

Current medical management options in patients with UCAs are limited, consisting largely of smoking cessation, blood pressure control, radiological surveillance, and neurosurgical or endovascular interventions. There is no pharmacological treatment available to decrease the risk of aneurysm growth and rupture. The current management of UCAs is controversial. There is an urgent need to understand the etiology of cerebral aneurysm and to establish reliable criteria by which surgeons can predict the risk of rupture and thus need for surgical intervention. There is growing interest in the pathogenesis of cerebral aneurysm focused on the development of drug therapies to decrease the incidence of aneurysm growth and rupture. Pathology-based therapies for patients harboring UCAs, especially in postmenopausal woman, are expected.

As previously mentioned, the pathology, etiology, and essential effector of cerebral aneurysm formation and progression are different in men and women and may also differ by age (i.e., those less than 40 years of age, those in their 40s–60s, and those older than 70). Based on these differences, it is possible that specific pharmacological treatment approaches may improve outcomes and the development of tailor-made medicines might be expected.

Although there are a number of factors that can potentially limit the translational potential of previously reported experimental findings, new-generation HRT may have a beneficial role in the prevention of aneurysm growth and rupture if initiated properly, that is, at the timing of perimenopause in women diagnosed with having small UCAs. Although the beneficial effect of this therapy in older women is less plausible because of preexisting cerebrovascular and cardiovascular risk factors, the continuation of this therapy through the menopausal period may have some benefit in older woman to some extent and should be elucidated in the future. Alternatives to traditional HRT, such as the use of highly selective ER-beta agonists and/or phytoestrogens, as well as the appropriate route of administration, dosage, timing, and choice of repetitive or periodic administration simulating the menstrual cycle (instead of chronic administration) could provide better approaches to increasing the benefits of HRT for prevention of aneurysmal growth and subsequent rupture causing SAH in postmenopausal women.

As sex differences remain an important issue in the development of cerebral aneurysm and SAH, further clinical studies are needed. An understanding of the relationship between estrogen and cerebral aneurysm/SAH pathophysiology may have significant implications for women's health.

## 3. Conclusion

There is growing interest in the pathogenesis of cerebral aneurysm focused on the development of drug therapies to decrease the incidence of aneurysm growth and rupture. Estrogen deficiency in postmenopausal women has a significant impact on the pathophysiology of cerebral aneurysm and SAH. Pathology-based therapies for patients harboring UCAs, especially in postmenopausal woman, seem to be a reasonable starting point for a disease with otherwise few treatment options and should be developed in the future.
